# A Rift Valley fever mRNA vaccine elicits strong immune responses in mice and rhesus macaques

**DOI:** 10.1038/s41541-023-00763-2

**Published:** 2023-10-27

**Authors:** Ting Bian, Meng Hao, Xiaofan Zhao, Chuanyi Zhao, Gang Luo, Zhendong Zhang, Guangcheng Fu, Lu Yang, Yi Chen, Yudong Wang, Changming Yu, Yilong Yang, Jianmin Li, Wei Chen

**Affiliations:** 1 Laboratory of Vaccine and Antibody Engineering, Beijing Institute of Biotechnology, Beijing, 100071 China; 2 Institute of Veterinary Medicine, Jiangsu Academy of Agricultural Sciences, Key Laboratory of Veterinary Biological Engineering and Technology, Ministry of Agriculture and Rural Affairs, Nanjing, China; 3 Frontier Biotechnology Laboratory, Zhejiang University-Hangzhou Global Scientific and Technological Innovation Center, Hangzhou, China; 4 School of Biotechnology, Jiangsu University of Science and Technology, Zhenjiang, China

**Keywords:** RNA vaccines, Virology

## Abstract

Rift Valley fever virus (RVFV) is listed as a priority pathogen by the World Health Organization (WHO) because it causes serious and fatal disease in humans, and there are currently no effective countermeasures. Therefore, it is urgent to develop a safe and efficacious vaccine. Here, we developed six nucleotide-modified mRNA vaccines encoding different regions of the Gn and Gc proteins of RVFV encapsulated in lipid nanoparticles, compared their ability to induce immune responses in mice and found that mRNA vaccine encoding the full-length Gn and Gc proteins had the strongest ability to induce cellular and humoral immune responses. IFNAR^(−/−)^ mice vaccinated with mRNA-GnGc were protected from lethal RVFV challenge. In addition, mRNA-GnGc induced high levels of neutralizing antibodies and cellular responses in rhesus macaques, as well as antigen-specific memory B cells. These data demonstrated that mRNA-GnGc is a potent and promising vaccine candidate for RVFV.

## Introduction

Rift Valley fever (RVF) is a mosquito-borne zoonotic disease caused by Rift Valley fever virus (RVFV), an important pathogen that causes substantial morbidity and mortality in both humans and animals^1,2^. Most infected patients develop a mild to moderate form of RVF disease that is characterized by a self-limiting, nonfatal, febrile illness. However, a small percentage of RVF patients develop severe symptoms, including hepatitis, retinitis, encephalitis, or hemorrhagic fever and the overall mortality rate is 0.5–1%^3,4^. Pregnant ruminants, especially sheep, are highly susceptible to RVFV infection and are typically subject to high-rates of abortions, fetal malformation, and febrile illness; newborn lambs usually show nearly 100% mortality^3,5^. Humans can become infected by the bite of an infected mosquito or contact with infected animal fluids or tissues^6^. Animal-to-animal and human-to-human transmission of RVFV has not been reported thus far, but vertical transmission has been demonstrated in both animals and humans^7–9^. In endemic areas, RVF outbreaks are often associated with weather events that result in excess rainfall, leading to flooding and subsequent mosquito blooms. RVF is now exclusively prevalent in Africa and the Arabian Peninsula^2^. However, due to the existence of risk factors such as extensive networks of global transportation, the presence of competent mosquito vectors, climate change and the large number of susceptible animals in nonendemic areas, RVF has the potential to spread in these areas^9,10^. RVF is considered a possible bioterror threat because it can be transmitted by aerosol, and there are currently no FDA-approved antiviral therapies or licensed vaccinations for humans^9^.

RVFV is a negative single-stranded RNA virus belonging to the order Bunyavirales and family Phenuiviridae^11^. Its genome is composed of three segments, small (S), medium (M), and large (L). The ambisense S segment encodes virus nucleoprotein (N) in the negative-sense orientation, which is required for RNA synthesis^12^, and the nonstructural (NSs) protein in the positive-sense orientation, which is the major virulence factor and functions to counteract the innate immune response by blocking the activation of the IFN-β promoter^13^. The M segment encodes the structural glycoproteins Gn and Gc, as well as the non-structural proteins NSm and a 78-kDa protein. The Gn and Gc glycoproteins are located on the surface of the virion as heterodimers, which then assemble into higher-order structures, and are involved in viral attachment and fusion, respectively. Studies have shown that the generation of neutralizing antibodies against Gn and Gc has provided a good correlate of protection in a variety of animals, such as mice, sheep, rhesus macaques, etc^9^. Therefore, these two proteins have been the main antigen targets in the development of RVF vaccines. NSm is suggested to function by inhibiting apoptosis^14^, and the 78-kDa protein is implicated in the transmission of RVFV from mosquitoes to ruminants, with a possible role in the replication of the virus in the mosquito host^15^. The L segment encodes viral RNA-dependent RNA polymerase (RdRP), which synthesizes both viral mRNA and genomic RNA^16^.

The low antigenic diversity and the presence of a single serotype are very beneficial for RVF vaccine development and disease control. Although many RVF vaccines have been licensed for veterinary use, these vaccines have suboptimal safety and efficacy^9,17^. Currently, there is no RVF vaccine that has been licensed for human use.

Compared with conventional vaccines, RNA vaccines represent a promising alternative because of their capacity to induce better immune response, rapid development, no risk of genomic integration, flexibility to respond to new variants, and safety when used for immunization^18,19^. In recent years, technological advances have allowed us to gradually overcome limitations that have hindered the application of mRNA, such as instability, excessive mRNA immunogenicity, and inefficient in vivo delivery. Multiple mRNA vaccines against infectious diseases have been developed and show encouraging results in both animal models and humans^20–24^. During the severe acute respiratory syndrome coronavirus 2 (SARS-CoV-2) pandemic, mRNA vaccines have been shown to be effective in protecting people from SARS-CoV-2 infection and were administered to millions of people around the world at an unprecedented rate to combat coronavirus disease 2019 (COVID-19). In addition, several mRNA vaccine candidates against other pathogens have entered clinical trials, including influenza virus^25^, rabies virus^26,27^, Zika virus^24^, RSV^28^ and so on^24,29^, indicating that mRNA vaccines are an extraordinary tool for combating emerging pandemics and existing infectious diseases. In this study, in order to investigate whether different regions of Gn and Gc proteins affect their abilities to induce immune response, six forms of mRNAs with different lengths of Gn and Gc proteins were constructed according to their structures^30,31^. After in vitro transcription using the modified nucleotide pseudouridine, the resulting mRNAs were packaged into lipid nanoparticles (LNPs). Then, the immunogenicity and protective efficacy of different mRNA vaccines were tested in mouse models, a candidate vaccine was selected, and its immunogenicity in rhesus macaques was evaluated.

## Results

### Immune responses induced by the designed mRNA vaccines in BALB/c mice

**Fig. 1:**
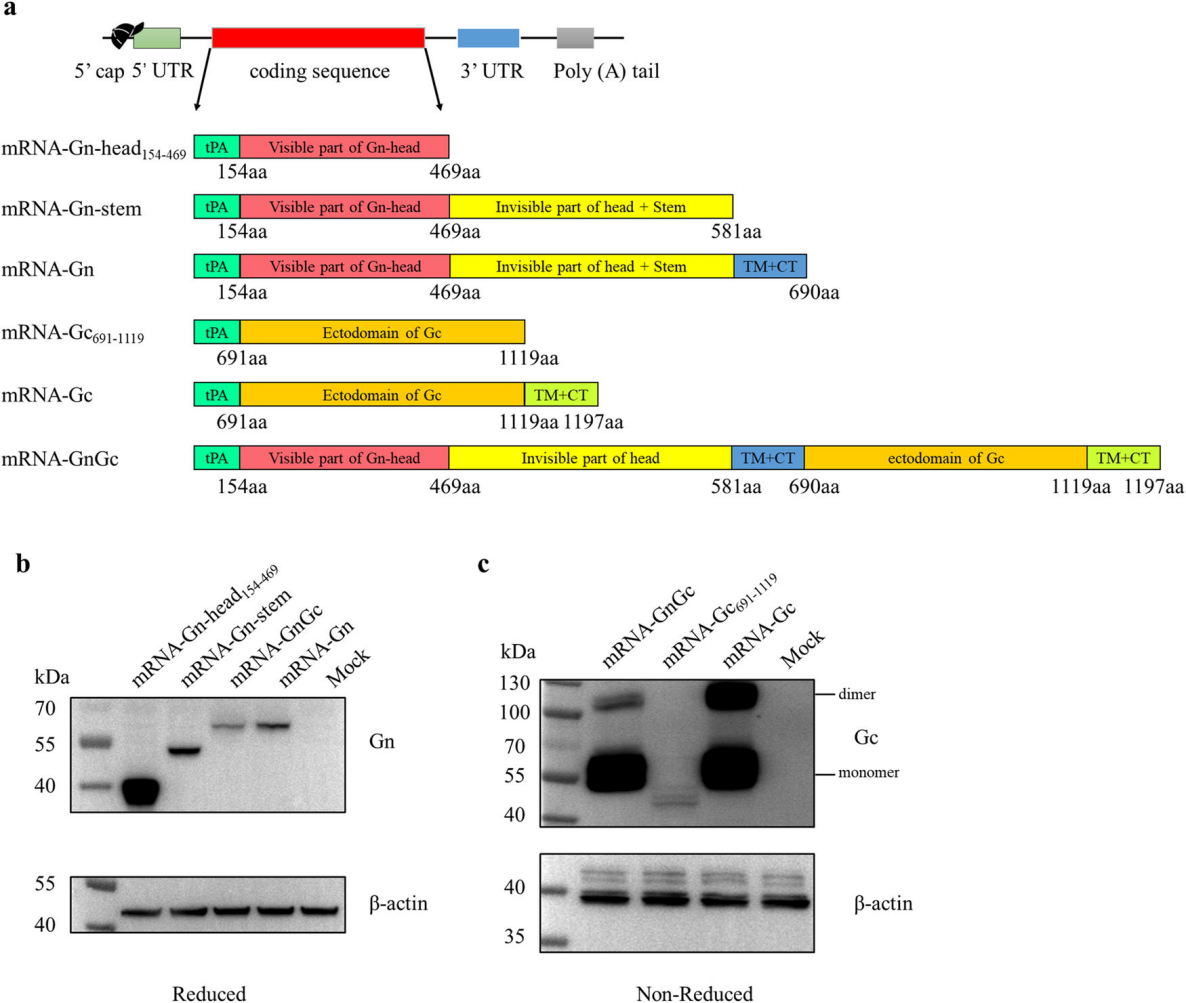
mRNA vaccines design and identification. **a** Schematic of the mRNA vaccines design. Six plasmids encoding different regions of RVFV Gn and Gc proteins were produced: (1) visible part of the Gn head from amino acids (aa) 154–469, (2) head and stem regions of Gn from amino acids 154–581, (3) full-length Gn from amino acids 154–690, (4) ectodomain of Gc from amino acids 691–1119, (5) full-length Gc from amino acids 691–1197, and (6) full-length Gn and Gc from amino acids 154–1197. The 5’ UTR was derived from human α-globin and the 3’UTR was a combination of human mitochondrial 12S rRNA and human AES/TLE5 gene, which were used in a SARS-CoV-2 mRNA vaccine^32^. TM Transmembrane, CT Cytoplasmic tail. Hela cells were transfected with mRNA-Gn-head_154-469_, mRNA-Gn-stem, mRNA-Gn, mRNA-Gc_691-1119_, mRNA-Gc, mRNA-GnGc (10 μg/well) and empty LNP, respectively. Western blotting under reducing conditions with a monoclonal antibody against RVFV Gn protein (**b**) or under non-reducing conditions with a monoclonal antibody against RVFV Gc protein (**c**) was performed at 48 h after transfection. Blots were derived from the same experiment and processed in parallel.

We engineered a series of plasmids encoding six forms of immunogens as follows: (1) visible part of the Gn head from amino acids 154 to 469, (2) head and stem regions of Gn, (3) full-length Gn, (4) ectodomain of Gc from amino acids 691 to 1119, (5) full-length Gc, and (6) full-length Gn and Gc. All the coding sequences contained a tissue plasminogen activator (tPA) signal peptide, and were flanked by the 5’ untranslated region (UTR, derived from human α-globin), the 3’UTR (a combination of human mitochondrial 12S rRNA and human AES/TLE5 gene) and the poly A tail (A30LA70), whose sequences were identical to a SARS-CoV-2 mRNA vaccine published previously^32^, to optimize translation efficiency and intracellular stability (Fig. 1a). During in vitro transcription, the modified nucleotide pseudouridine was used instead of uridine to dampen indiscriminate immune activation, which might inhibit mRNA translation, thereby reducing antigen expression and the immunogenicity of an mRNA vaccine^33,34^. After transcription, a 5’ cap-1 structure was enzymatically added to produce fully mature mRNA. The expression profiles of different mRNAs were characterized by transfecting Hela cells. For the monoclonal antibody we have against Gn protein recognizes linear epitope, the expression of Gn protein was identified by western blotting under reducing condition. And the results showed that Gn proteins of different sizes were well expressed by related mRNA designs, with mRNA-GnGc having the weakest expression level (Fig. 1b and Supplementary Fig. 1a). Monoclonal antibody we have against Gc protein recognizes conformational epitope, so the expression of Gc protein was identified under non-reducing condition. And the results showed that the expression of Gc protein could be detected in all related mRNA designs. It should be noted that Gc protein expressed by mRNA-Gc_691-1119_ could not form a complete conformational epitope recognized by the monoclonal antibody, resulting in a weakened antibody binding ability and weak detection bands (Fig. 1c and Supplementary Fig. 1b). To shield mRNA from extracellular RNases and ensure the efficiency of delivery into cells, the purified mRNAs were encapsulated in lipid nanoparticles^35^. The encapsulation efficacy of mRNAs was >90%, as determined by a RiboGreen RNA quantification assay.

**Fig. 2:**
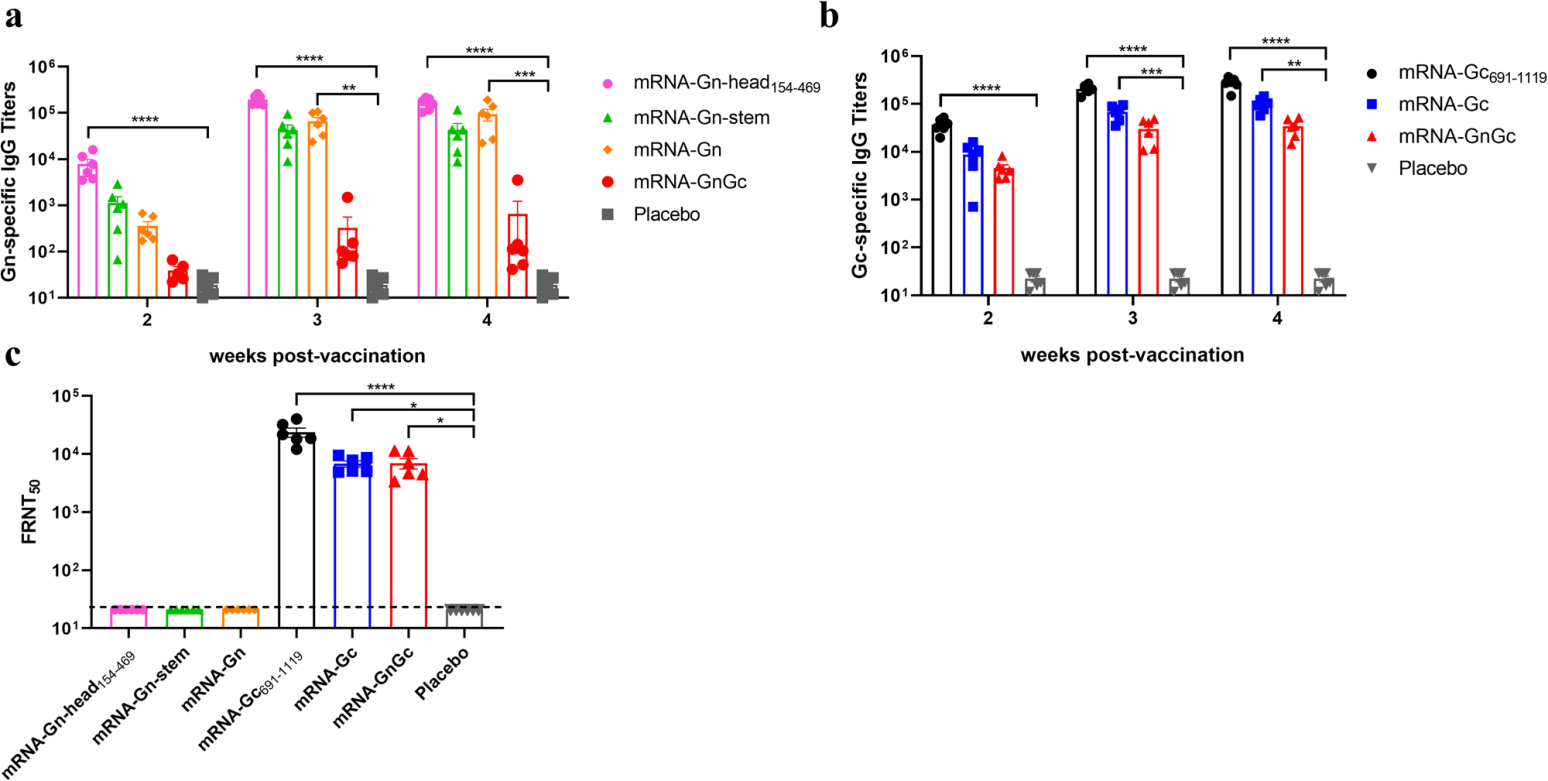
Humoral immune response induced by different mRNA vaccines in mice. Groups of BALB/c mice (*n* = 6) were immunized intramuscularly with 5 μg of mRNA-Gn-head_154-469_, mRNA-Gn-stem, mRNA-Gn, mRNA-Gc_691-1119_, mRNA-Gc, mRNA-GnGc or empty LNP as placebo and boosted with the same dose on day 14. Sera were collected 14, 21, and 28 days after initial vaccination and subjected to antibody detection. **a** RVFV Gn-specific IgG antibody titers were determined by ELISA. **b** RVFV Gc-specific IgG antibody titers. **c** Neutralizing antibody titers in sera collected two weeks after booster immunization were measured by a FRNT_50_. Data are shown as the mean ± SEM. *P* values were calculated by one-way ANOVA with multiple comparison tests. *P < 0.05, **P < 0.01, ***P < 0.001, ****P < 0.0001.

To test the immunogenicity and efficacy of these mRNA-LNP vaccines in animals, groups of female BALB/c mice (*n* = 6) were immunized intramuscularly with 5 μg of mRNA-Gn-head_154-469_, mRNA-Gn-stem, mRNA-Gn, mRNA-Gc_691-1119_, mRNA-Gc, mRNA-GnGc or empty LNP as placebo and boosted with the same dose on day 14. Sera were collected 14, 21, and 28 days after initial vaccination and subjected to antibody detection. ELISA results showed that all the mRNA-LNPs could induce Gn- or Gc-specific IgG responses, and a second immunization resulted in a rapid elevation of antibody titers, with the highest titer in the mRNA-Gn-head_154-469_ and mRNA-Gc_691-1119_ groups and the lowest titer in the mRNA-GnGc group, which are presumably caused by the different molar amounts of antigen among these groups (Fig. 2a, b). Sera collected 28 days after initial immunization were used to determine the neutralizing antibody (Nab) titers with the rescued reporter virus rMP-12-eGFP by a 50% focus reduction neutralization test (FRNT_50_) as described previously^36^. The results showed that groups mRNA-Gc_691-1119_, mRNA-Gc and mRNA-GnGc all presented much higher titers of neutralizing antibody, among which group mRNA-Gc_691-1119_ was the highest, while no neutralizing antibody was detected in groups mRNA-Gn-head_154-469_, mRNA-Gn-stem and mRNA-Gn (Fig. 2c). No Gn- and Gc-specific IgG and neutralizing antibodies were detected in sera from mice vaccinated with placebo.

**Fig. 3:**
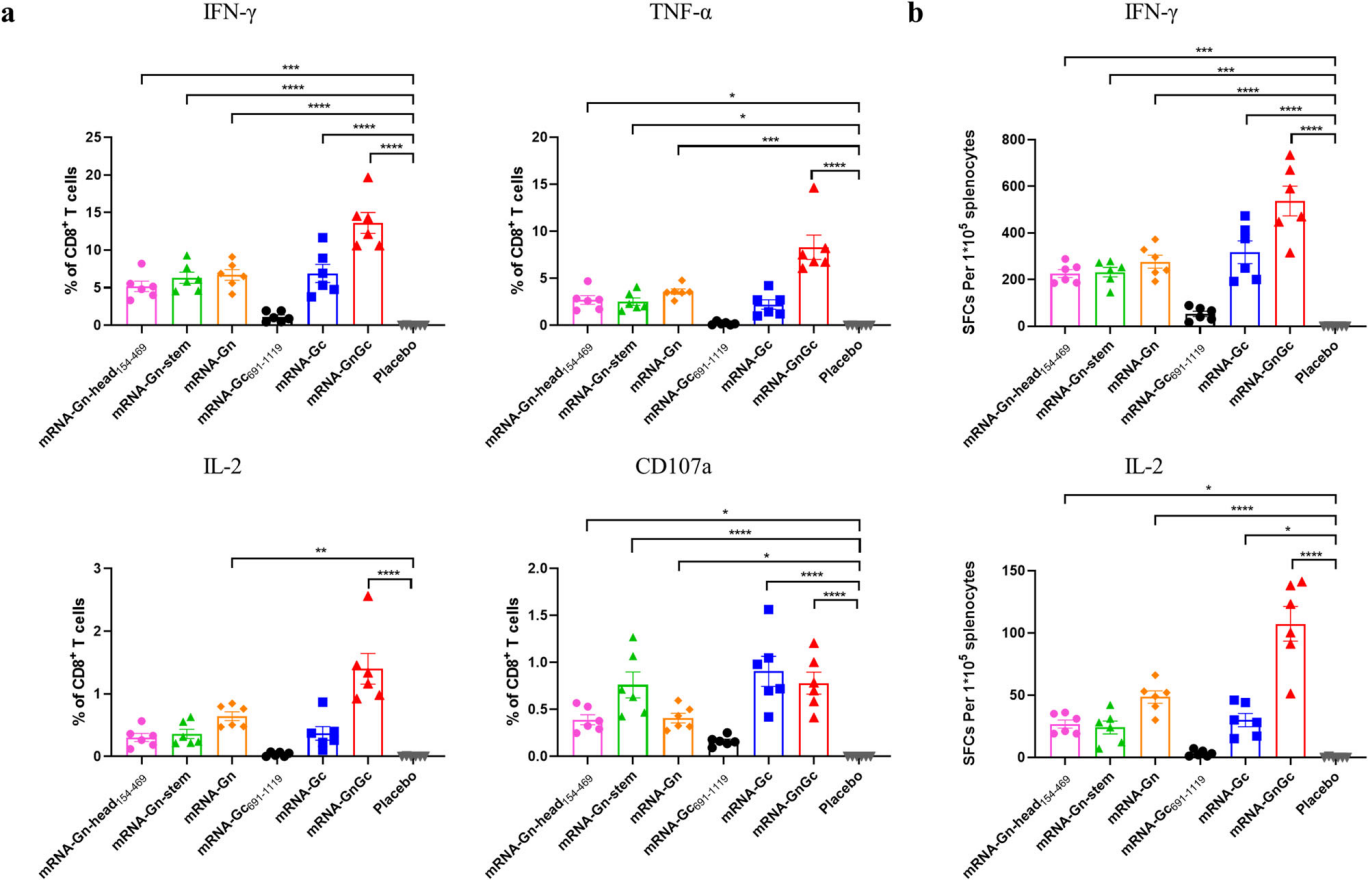
RVFV-specific T-cell immune response induced by different mRNA vaccines in mice. Groups of BALB/c mice were immunized intramuscularly with 5 μg of mRNA-Gn-head_154-469_, mRNA-Gn-stem, mRNA-Gn, mRNA-Gc_691-1119_, mRNA-Gc, mRNA-GnGc or a placebo and boosted with an equivalent dose 14 days later. Two weeks after booster immunization, the mice were sacrificed, and splenocytes were collected. **a** The percentages of CD8^+^ T cells secreting IFN-γ, TNF-α, IL-2 or CD107a were determined by ICS. **b** ELISPOT assay was performed to assess IFN-γ and IL-2 secretion by mouse splenocytes. Data are shown as the mean ± SEM. *P* values were calculated by one-way ANOVA with multiple comparison tests. *P < 0.05, **P < 0.01, ***P < 0.001, ****P < 0.0001.

We further studied whether an RVFV-specific T-cell immune response was elicited by two doses of these mRNA-LNP vaccines in BALB/c mice. Splenocytes from vaccinated mice were harvested 2 weeks after booster immunization and tested with intracellular cytokine staining (ICS) and ELISPOT assays. The results showed that the proportions of CD8^+^ T cells that secreted IFN-γ, TNF-a and IL-2 were all significantly increased in the vaccine-immunized groups except in the mRNA-Gc_691-1119_ group; the mRNA-GnGc group had the highest proportion, which was significantly higher than that of the other groups (Fig. 3a, b). In addition, the number of CD8^+^ T cells that secreted CD107a, a marker of cytotoxic CD8^+^ T-cell degranulation and cytotoxic activity, was also significantly increased in the vaccine-immunized groups (Fig. 3a). As expected, no corresponding response was detected in the control group.

**Fig. 4:**
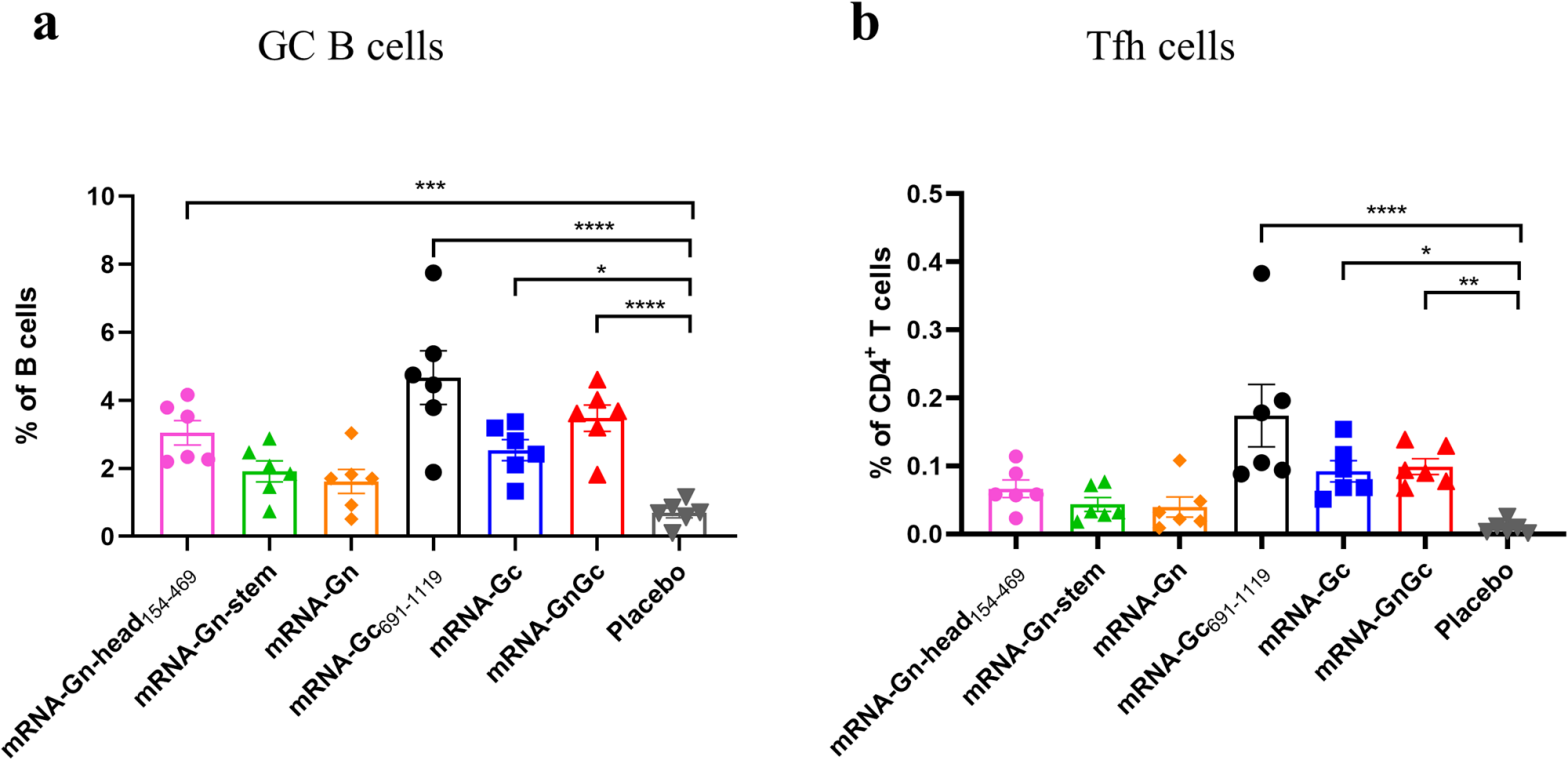
GC B cell and Tfh cell responses induced by different mRNA vaccines in mice. Groups of BALB/c mice (*n* = 6) were immunized intramuscularly with 5 μg of mRNA-Gn-head_154-469_, mRNA-Gn-stem, mRNA-Gn, mRNA-Gc_691-1119_, mRNA-Gc, mRNA-GnGc or a placebo and boosted with the same dose at a 14-day interval. Ten days after booster immunization, the mice were sacrificed, and the inguinal lymph nodes were collected. **a** The proportions of GC B cells (CD3^-^CD19^+^GL7^+^Fas^+^) were determined by flow cytometry. **b** The frequency of Tfh cells (CD3^+^CD4^+^PD-1^+^CXCR5^+^) was measured in each group. Data are displayed as the mean ± SEM. *P* values were calculated by one-way ANOVA with multiple comparison tests. *P < 0.05, **P < 0.01, ***P < 0.001, ****P < 0.0001.

As the important microanatomical sites of B-cell mutation and antibody affinity maturation^37,38^, we also determined the germinal centers (GCs) responses after these mRNA-LNP vaccine immunizations. Groups of BALB/c mice (*n* = 6) were immunized intramuscularly with 5 μg above vaccines and boosted with the same dose at a 14-day interval. The inguinal lymph nodes were collected 10 days after booster immunization and subjected to GC B cell and T follicular helper (Tfh) cell detection by flow cytometry. Our results showed that the percentages of total GC B cells and Tfh cells were all significantly increased in the mRNA-Gc_691-1119_, mRNA-Gc, and mRNA-GnGc groups but not in the mRNA-Gn-head_154-469_, mRNA-Gn-stem and mRNA-Gn groups (Fig. 4a, b), which was consistent with the results of the neutralizing antibody experiments. However, the reason for the inconsistency with the overall antibody levels needs further exploration. Taking all the above experimental results into consideration, we selected mRNA-GnGc as the vaccine candidate for further research.

### Protection efficacy of mRNA-GnGc in IFNAR^(−/−)^ mice against RVFV

**Fig. 5:**
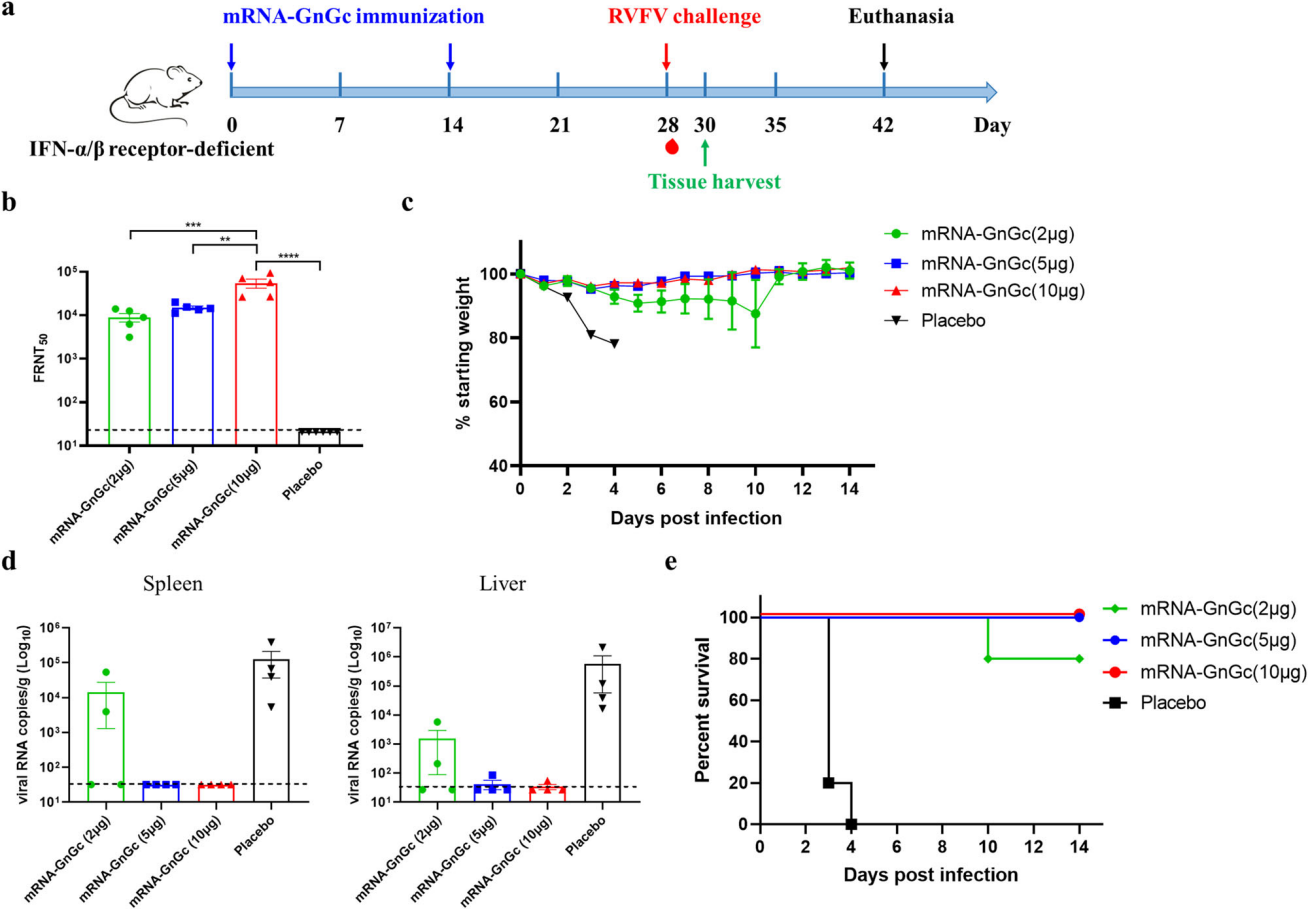
Protection of candidate vaccine mRNA-GnGc against RVFV challenge in IFNAR^(−/−)^ mice. Mice (*n* = 9) received two doses of 2 μg, 5 μg or 10 μg of mRNA-GnGc or a placebo at a 14-day interval via the intramuscular route. Two weeks after booster vaccination, the mice were challenged intraperitoneally with 2 × 10^4^ TCID_50_ of the RVFV rMP-12 strain. Two days after challenge, four mice from each group were selected, and their livers and spleens were collected for viral RNA load detection. **a** Schematic diagram of the immunization and challenge schedule. **b** Four weeks after the initial vaccination (before challenge), serum neutralizing antibody titers were determined using infectious RVFV. **c** Mouse body weight changes after RVFV challenge. **d** Viral loads in the spleen and liver two days after challenge were determined by qRT–PCR. **e** Mortality and survival curves of mice. Data are displayed as the mean ± SEM. *P* values were calculated by one-way ANOVA with multiple comparison tests. **P < 0.01, ***P < 0.001, ****P < 0.0001.

IFNAR^(−/−)^ mice lacking type I interferon α/β receptor are permissive to a lethal RVFV challenge and are frequently used for the evaluation of the protective efficacy of the vaccine^36,39,40^. To further explore the in vivo protection efficacy of mRNA-GnGc against RVFV challenge, groups of IFNAR^(−/−)^ mice (n = 9) immunized intramuscularly with two doses of 2 μg (low-dose), 5 μg (middle-dose), 10 μg mRNA-GnGc (high-dose) or placebo at a 14-day interval were challenged intraperitoneally with 2 × 10^4^ median TCID_50_ of the RVFV rMP12 strain 2 weeks after booster vaccination. The pre-challenge sera were collected for detection of NAb titers (Fig. 5a). The results showed that all the three groups generated high levels of NAbs in a dose-dependent manner, as determined by live virus neutralization assays (Fig. 5b). After challenge, the body weight of each mouse was recorded daily for 14 days, and the results showed that mice in the placebo group decreased significantly, with 16–22% body weight loss, while mice in the middle-dose and high-dose groups displayed mild weight loss at 1 to 6 days post infection (dpi), followed by a rapid increase to normal levels at 7–14 dpi. One mouse in low-dose group decreased significantly and succumbed 10 days after challenge, while the other four mice displayed a little weight loss at 1–8 dpi and then a rapid increase to a normal level from 9 to 14 dpi (Fig. 5c). Four mice from each group were euthanized two days after challenge, and spleen and liver tissues were harvested for viral RNA load detection. Our results showed that tissues from the middle-dose and high-dose groups showed substantially reduced infectious virus burden with almost no viral RNA detectable. In the low-dose group, viral RNA was detected in the livers and spleens of two mice (Fig. 5d). During the whole experiment, all vaccinated mice in the middle-dose and high-dose groups survived the challenge. One mouse in the low-dose group showed late-onset weight loss and succumbed at 10 days post-challenge, while all the other mice survived. However, mice in the placebo group suffered significant and rapid weight loss, and all succumbed to infection 3–4 days after the challenge (Fig. 5c, e). These data demonstrated the high efficacy of mRNA-GnGc in a lethal mouse RVFV challenge model.

### Immune responses induced by mRNA-GnGc in rhesus macaques

**Fig. 6:**
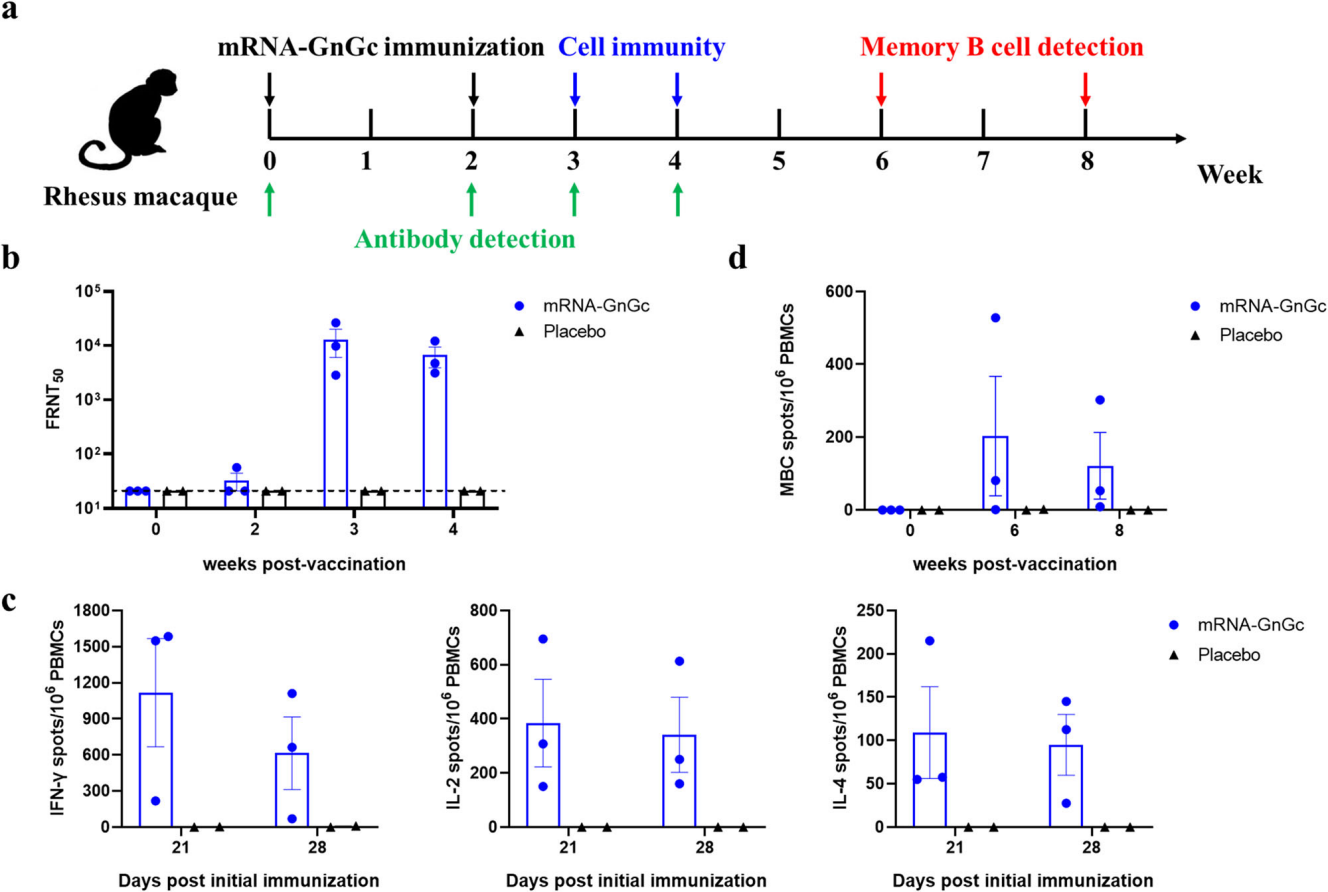
Immunogenicity evaluation of mRNA-GnGc in rhesus macaques. Five healthy rhesus macaques (2 females, 3 males, between 5–6 years old) were randomly assigned into two groups and immunized intramuscularly with 100 μg of mRNA-GnGc (*n* = 3) or placebo (*n* = 2) and boosted with the same dose at a 14-day interval. **a** Schematic diagram of mRNA-GnGc immunization, sample collection and immunological assays. **b** Neutralizing antibody titers in serum collected on days 0, 14, 21 and 28 after initial immunization were measured by a 50% focus reduction neutralization test. **c** PBMCs collected 21 and 28 days after initial immunization were stimulated with RVFV Gn and Gc peptide pools, and the secretion of IFN-γ, IL-2 or IL-4 was measured by an ELISPOT assay. **d** An ELISPOT assay was performed to assess the number of antigen-specific MBCs in PBMCs collected on weeks 0, 6 and 8 after initial vaccination. Data are displayed as the mean ± SEM.

To investigate the immunogenicity of this newly designed vaccine in rhesus macaques, five macaques were divided into two groups, with three in the vaccine-immunized group and two in placebo group. Two groups of animals were immunized twice (on day 0 and day 14) with 100 μg of mRNA-GnGc or placebo via intramuscular administration (Fig. 6a). Our results showed that neutralizing antibodies were almost undetectable 14 days after the initial immunization, whereas booster immunization resulted in a notable increase in FRNT_50_, with FRNT_50_ values of ~13,000 and ~6600 at three and four weeks after the initial immunization, respectively, indicating that mRNA-GnGc could induce a strong humoral immune response (Fig. 6b). To determine if the candidate vaccine could also elicit a cellular immune response in rhesus macaques, peripheral blood mononuclear cells (PBMCs) were collected and stimulated with a peptide pool for RVFV Gn and Gc proteins. ELISPOT assay results showed that T cells secreting IFN-γ, IL-2, and IL-4 from mRNA-GnGc-immunized macaques were more numerous than those from the placebo-immunized group (Fig. 6c). In addition, to investigate the frequency of memory B cells (MBCs) in rhesus macaques after immunization with mRNA-GnGc, an ELISPOT assay was developed to quantify antigen-specific MBCs as a readout of humoral immune memory. PBMCs collected from immunized animals six and eight weeks after the initial vaccination were stimulated with R848 and human IL-2 for 4 days, which was optimized to drive the transition of memory B cells to antibody-secreting cells (ASCs). Then, the frequency of antigen-specific ASCs could be measured by ELISPOT assay. Our results showed that antigen-specific MBCs could be detected in two out of three animals with relatively large individual differences (Fig. 6d), which indicated that mRNA-GnGc had the ability to elicit antigen-specific memory B-cell responses that should prolong immunity.

## Discussion

Large outbreaks of RVFV can have a devastating impact on human and animal health. Vaccination is the most effective and economical intervention to control the spread of epidemics, thereby saving lives and protecting people’s health, which was best demonstrated during the COVID-19 pandemic, when a large number of vaccines were developed to control the spread of the epidemic. Among these, mRNA vaccines have attracted much more attention due to their great application prospects and advantages, such as a short development cycle, easy industrialization, simple production process, flexible response to new variants, and strong capacity to induce an immune response. In the *phlebovirus* field, no experimental mRNA vaccine has been developed. Here, we developed six modified mRNAs encoding different regions of Gn and Gc proteins of RVFV encapsulated in lipid nanoparticles and demonstrated that the candidate vaccine mRNA-GnGc could offer high protection against RVFV.

As glycoproteins, Gn and Gc are the main proteins where neutralizing epitopes exist, they are ideal targets for vaccine development and the screening of neutralizing antibodies^40–44^. Most of the previous studies chose Gn protein or Gn and Gc proteins as the target antigen. Furthermore, few studies have reported the ability of individual Gc protein to induce immune response. In this study, mRNA vaccines expressing different regions of RVFV Gn and Gc proteins were designed according to their structures to compare the differences in their ability to induce an immune response. Our results showed that all six mRNA vaccines could induce the production of antigen-specific binding antibodies. However, to our surprise, three designs, mRNA-Gn-head_154-469_, mRNA-Gn-stem and mRNA-Gn, failed to induce neutralizing antibodies, while the remaining three designs, mRNA-Gc_691-1119_, mRNA-Gc and mRNA-GnGc, were able to induce high levels of neutralizing antibodies. The differences in neutralizing antibody titers might be caused by the different molar amounts of antigen or different epitope presentation abilities among these three groups. In line with our finding, Chrun et al. constructed a DNA vaccine encoding the extracellular portion of the Gn antigen and found that it was unable to induce neutralizing antibody production in sheep^45^. Similarly, a DNA vaccine encoding the untargeted ectodomain of the Gn protein constructed by Bhardwaj et al. only minimally induced neutralizing antibodies in mice^46^. In addition, the soluble ectodomain of the Gn protein produced by Drosophila cells and formulated in Stimune water-in-oil adjuvant could induce neutralizing antibodies in sheep, but the titer was very low^47^. Of course, vaccines based on Gn protein alone could induce high titers of neutralizing antibody were also reported^48^. The reasons beneath this phenomenon are worth further investigation. The ability of vaccines expressing Gc protein alone to induce an immune response is rarely reported. Our results show that high levels of neutralizing antibody in mice could be induced by mRNA vaccines expressing the ectodomain (mRNA-Gc_691–1119_) or the full-length Gc protein (mRNA-Gc). In addition, an mRNA vaccine expressing Gn and Gc proteins simultaneously (mRNA-GnGc) can also induce high levels of neutralizing antibodies in both mice and rhesus macaques. However, paradoxically, studies on neutralizing antibodies against RVFV have shown that most of the antibodies with good neutralizing activity are against the Gn protein, while the number of neutralizing antibodies against the Gc protein is small, and their neutralizing activity is poor^42,43^. We speculated that the above contradictory phenomena might be caused by the different conformations of the Gn protein produced by different expression systems, resulting in different abilities to induce neutralizing antibody production. However, this phenomenon still needs to be verified by investigating more expression systems that express these three antigens. Meanwhile, structural analysis can be used to explore the molecular characteristics of the three antigenic proteins in different expression systems.

Although mRNA-Gn-head_154-469_, mRNA-Gn-stem and mRNA-Gn failed to induce neutralizing antibody production, they induced strong cellular immune responses. Another unexpected finding is that there is a significant difference between the ability of mRNA-Gc_691-1119_ and mRNA-Gc to induce the cellular immune response. mRNA-Gc can induce a strong cellular immune response, while mRNA-Gc_691-1119_ can only barely induce the corresponding response. We speculated that this phenomenon may be caused by the fact that most Gc proteins synthesized by mRNA-Gc are distributed intracellularly, so it is easier for the Gc proteins to be processed and presented by major histocompatibility complex (MHC) class I molecules, which activates the MHC class I antigen presentation pathway and induces antigen-specific cellular immune responses.

Considering the ability of the six vaccines to induce cellular and humoral immune responses, we selected mRNA-GnGc as the candidate vaccine to evaluate protective activity in mice. IFNAR^(−/−)^ mice are susceptible to many viruses and are often used as animal models to evaluate antiviral drugs and vaccines for viral infections, such as Zika virus or RVFV^39,40,49^. In the present research, we evaluated the protective immune response induced by mRNA-GnGc in this animal model. The results of RVFV challenge experiment showed that all mice in the LNP group died 3–4 days after challenge, while low-dose vaccine immunization significantly reduced clinical signs and improved the survival rate of mice. Only one out of five mice died, and the time of death was significantly delayed. Mice in the middle-dose and high-dose groups showed no obvious clinical signs except for temporary weight loss, and all survived with almost no virus detected in the tissues, indicating that the candidate vaccine mRNA-GnGc could significantly inhibit the proliferation of RVFV in mice and provide good protective efficacy for animals. However, it needs to be noted that due to the lack of wild-type virus, the rescued virus rMP-12 was used instead of wild-type RVFV MP-12 based on their similar growth kinetics^50^. In addition, the reporter virus rMP-12-eGFP was also obtained and used in the microneutralization assay, the growth kinetics of the reporter virus were similar to those of rMP-12, as reported by previous research^50^. Another limitation of our study was that the challenge experiments were based on the attenuated RVFV strain MP-12 and IFNAR^(−/−)^ mouse model, which cannot fully reflect the protective properties of the vaccine against virulent RVFV infection in immunocompetent mice. Further challenge experiments with a virulent strain and immunocompetent mice will make the data on protective efficacy more comprehensive.

The ability of mRNA-GnGc to induce immune responses in rhesus macaques, an animal species that has been used extensively as a surrogate model for RVF disease in humans^51–53^, was also evaluated. Our results showed that the candidate vaccine could induce neutralizing antibody production and antigen-specific cellular immune responses in rhesus macaques. In addition, antigen-specific memory B cells were also detected in two of the three animals. However, due to the limitation of experimental conditions, the number of rhesus monkeys was small, and the individual differences were relatively large, so the ability of mRNA-GnGc to induce an immune response still needs to be evaluated in more animals.

In conclusion, we compared the differences in the ability of mRNA vaccines expressing different regions of Gn and Gc proteins to induce immune responses and found that mRNA vaccines expressing the full-length Gn and Gc proteins had the strongest ability to induce an immune response. This vaccine can inhibit the proliferation of RVFV in mice, protect mice from RVFV pathogenesis, and induce a relatively strong immune response in rhesus macaques, indicating that mRNA-GnGc is a promising candidate vaccine against RVFV. Further work is needed to confirm these results in a larger study and to elucidate the initiation and duration of immunity.

## Methods

### Ethics statement

All animal experimental protocols were approved by the Animal Care and Use Committee of Beijing Institute of Biotechnology, China (Permit number for mouse experiments: IACUC-SWGCYJS-2021-006, Permit number for rhesus macaque experiments: E20220615) in strict accordance with the Guide for the Care and Use of Laboratory Animals of the People’s Republic of China.

### Cells, viruses, and animals

Vero E6 cells and Hela cells were grown in Dulbecco’s modified Eagle’s medium (DMEM) supplemented with 100 U/mL penicillin, 100 mg/mL streptomycin, and 10% heat-inactivated foetal bovine serum (FBS, Gibco) at 37 °C and 5% CO_2_. The rescued viruses rMP-12 and rMP-12-eGFP were prepared as previously described^36,50^, propagated in Vero E6 cells, and stored at −80 °C.

Female BALB/c mice (specific pathogen-free), aged 6–8 weeks, used in this study were purchased from SPF (Beijing) Biotechnology Co., Ltd. (Beijing, China) and bred in the animal facility of the Animal Center, Beijing Institute of Biotechnology. Interferon-α/β receptor-deficient (IFNAR^(−/−)^) A129 mice were preserved and housed in the animal facility of the Animal Center, Beijing Institute of Biotechnology. The animal experiments involving RVFV challenge were conducted in the animal biosafety level 2 (ABSL2) facilities of the Beijing Institute of Biotechnology.

Five healthy rhesus macaques (2 females, 3 males, between 5–6 years old) used for vaccine immunogenicity analysis were purchased and housed at the Beijing Institute of Xieerxin Biology Resource.

### Vaccine design and mRNA-LNP preparation

According to the structures of the RVFV glycoproteins Gn and Gc^30,31^, six constructs encoding different regions of Gn and Gc proteins based on the MP-12 strain (accession number DQ380208.1) were codon optimized for human cells by biology software and synthesized by General Biol (Anhui) Co., Ltd. All the constructs contained a T7 promotor, a tPA signal peptide, a 5’UTR, a 3’UTR and a 100 nucleotide poly(A) tail interrupted by a linker (A30LA70, 10 nucleotides). The sequences of the 5’ and 3’UTRs were identical to those in a previous publication with a SARS-CoV-2 mRNA vaccine^32^. mRNA transcripts were synthesized in vitro from linearized DNA templates by using a T7 High Yield RNA Transcription Kit, where the UTP was substituted with 1-methylpseudo UTP to generate modified nucleoside-containing mRNA (Vazyme). The 5’ cap-1 structure was enzymatically added to increase mRNA translation efficiency (Vazyme). mRNA was encapsulated in lipid nanoparticles as previously described^54,55^. The encapsulation efficiency and mRNA concentration of mRNA-LNPs were measured using Quant-iT RiboGreen RNA Reagent and Kit (Invitrogen).

### mRNA transfection and identification

1 × 10^6^ Hela cells were seeded in six-well plates. Eighteen hours later, the cells were transfected with mRNA transcripts (10 μg/well) using Lipofectamine 3000 Transfection Reagent (Thermo Fisher Scientific) according to the manufacturer’s protocol. Forty-eight hours after transfection, the cells were washed with phosphate-buffered saline (PBS) pH 7.4 and lysed with RIPA lysis buffer (Thermo Fisher Scientific) for twenty minutes on ice. After centrifugation, the supernatant was collected and mixed with loading buffer with or without dithiothreitol and separated by 10% SDS-PAGE. The separated proteins transferred to polyvinylidene fluoride membranes was performed by an eBlot L1 transfer system (GenScript, China). The membrane was blocked with 5% non-fat milk in PBS buffer. Gn and Gc proteins were detected using anti-Gn (at a final concentration of 1 µg/mL) and anti-Gc monoclonal antibody (at a final concentration of 1 µg/mL), followed by secondary antibodies labeled with HRP (at a final concentration of 0.1 µg/mL, Abcam). The membranes were developed with a chemiluminescent substrate (Merck Millipore), and images were acquired with an iBright 1500 imaging system (Thermo Fisher Scientific).

### Animal experiments

LNP-encapsulated mRNA transcripts were diluted with PBS. Groups of female BALB/c mice aged 6–8 weeks were inoculated intramuscularly with different immunogens (5 μg) or empty LNP as placebo in 100 μL using insulin syringes (BD Biosciences) and boosted with equal doses on day 14 post initial immunization. Serum samples were collected at the indicated times after vaccination, inactivated at 56 °C for 30 min, and used for Gn- and Gc-specific IgG and anti-RVFV neutralizing antibody titration. The inguinal lymph nodes were collected ten days after booster immunization to evaluate germinal center responses. Spleen tissues were collected two weeks after booster immunization for evaluation of cellular immune responses by ELISPOT and flow cytometry as described below.

For the RVFV challenge experiment, groups of IFNAR^(−/−)^ mice (*n* = 9) were vaccinated intramuscularly at 0 and 14 days with different immunogens or placebo. Two weeks after the second immunization, animals were bled and challenged intraperitoneally with 2 × 10^4^ TCID_50_ of the RVFV rMP-12 strain and then monitored for survival. Two days after challenge, mice (*n* = 4) from each group were euthanized, and livers and spleens were collected to determine RVFV loads. During the whole challenge experiment, mice were evaluated daily for clinical signs of disease and anesthetized by intraperitoneal injection of sodium pentobarbital and euthanized by cervical dislocation when clinical scores reached humane endpoint (8 point) according to a predetermined clinical illness scoring algorithm^40,56,57^.

To evaluate the immunogenicity of mRNA-GnGc in nonhuman primates, a total of five rhesus macaques were randomly assigned into two groups, with three in the vaccine-immunized group and two in the placebo group, and immunized intramuscularly with mRNA-GnGc (100 μg) or placebo two times at a 14-day interval. PBMCs and serum samples were collected at the indicated time points for the detection of cytokines, memory B cells and neutralizing antibodies.

### Protein expression and purification

The RVFV Gn head domain (accession number DQ380208.1, residues 154–469) and Gc ectodomain (residues 691–1119) were subcloned into pCAGGs vector respectively. The C-terminal of both plasmids were labeled with a strep-tag II. After transfection into Expi293F mammalian suspension cells for four days, the supernatants were harvested and purified by affinity chromatography with a 5-mL StrepTrap^TM^ HP column according to the manufacturer’s protocol (GE Healthcare).

### Enzyme-linked immunosorbent assays (ELISA)

The 96-well plates (Corning) were coated overnight with 2 μg/mL RVFV Gn or Gc protein in carbonate–bicarbonate buffer (pH 9.6) at 4 °C. Plates were washed with PBS containing 0.2% Tween-20 (PBST) and blocked with 2% BSA in PBS at 37 °C for 1 h the next day. Serum samples from immunized animals were serially diluted and added to the blocked plates. After incubation at 37 °C for 1 h, plates were washed with PBST and incubated with goat anti-mouse IgG (cat. number ab6789, 1:20,000, Abcam) or goat anti-monkey IgG-HRP (cat. number ab112767, 1:20,000, Abcam) at 37 °C for 1 h. Then, the plates were washed with PBST, and TMB substrate solution (Solarbio, China) was added. The reactions were stopped by 2 M sulfuric acid. The absorbance at 450 nm/630 nm was read using a microplate reader (SPECTRA MAX 190, Molecular Device). The endpoint titers were defined as the reciprocal of the highest serum dilution that produced an optical absorbance value 2.1-fold higher than the optical absorbance value of the negative control.

### Focus reduction neutralization assay (FRNT)

The neutralizing activity of sera from immunized animals was assessed using an RVFV microneutralization assay. Briefly, heat-inactivated serum samples were serially diluted and incubated with rMP-12-eGFP (100 TCID_50_) in 96-well plates for 1 h at 37 °C. Subsequently, Vero E6 cells were added to each well. After incubation for 48 h at 37 °C, cells were fixed with 4% formaldehyde for 2 h and then counterstained with DAPI (1:1000, Thermo Fisher Scientific). The number of infected cells (eGFP) and total cells (DAPI) were measured by a Celigo (Nexcelom) imaging cytometer. The reciprocal of the serum dilution at which 50% of foci were neutralized is reported as the FRNT_50_.

### ICS

To evaluate cytokine expression in antigen-specific T cells, an ICS assay was performed. The spleens from immunized mice were collected and homogenized into a single-cell suspension. Then the splenocytes were seeded in 24-well plates (2 × 10^6^ cells/well) and stimulated with fourteen peptides derived from RVFV Gn and Gc proteins (2 µg/mL) identified previously^58^, together with BD GolgiStop™ (BD Biosciences) for 8 h at 37 °C. The cells were harvested, incubated in live/dead near IR (Thermo Fisher) and blocked with anti-CD16/32 (cat. number 156604, 1:150, BioLegend). Following a wash in PBS, cells were stained for 30 min with a mixture of anti-mouse antibodies purchased from BioLegend, including CD3 PerCP/Cyanine5.5 (cat. number 10021817, 1:150), CD4 Alexa Fluor 700 (cat. number 100536, 1:250), CD19 APC/Cyanine7 (cat. number 115530, 1:250), CD8a Brilliant Violet 510™ (cat. number 100751, 1:150), and CD107a Brilliant Violet 421™ (cat. number 121617, 1:100). After a wash in PBS, cells were fixed and permeabilized with Cytofix/Cytoperm (BD Biosciences), washed with Perm/Wash buffer (BD Biosciences), and stained for 30 min with a mixture of anti-mouse antibodies purchased from BioLegend, including IFN-γ PE (cat. number 505808, 1:100), TNF-α Alexa Fluor 647 (cat. number 506314, 1:100) and IL-2 PE/Cyanine7 (cat. number 503832, 1:100). Finally, cells were washed and resuspended in PBS prior to the acquisition of data on a FACS Canto™ flow cytometer (BD Biosciences). Gating strategies are showed in Supplementary Fig. 2.

### Flow cytometry

To evaluate the germinal center responses in vaccine-immunized mice, the draining inguinal lymph nodes were collected and homogenized into a single-cell suspension. Cells were stained with live/dead near IR (Thermo Fisher) and Fc blocked with anti-CD16/32 monoclonal antibody (cat. number 156604, 1:150, BioLegend) prior to staining. Then, the cells were stained with a mixture of monoclonal antibodies, including anti-CD19 Brilliant Violet 605™ (cat. number 115539, 1:400), anti-GL7 PE (cat. number 144608, 1:150), anti-Fas AF647 (cat. number 152620, 1:200), anti-CD4 FITC (cat. number 100510, 1:250), anti-PD-1 Brilliant Violet 421™ (cat. number 135217, 1:50), anti-CXCR5 PE/Cyanine7 (cat. number 145516, 1:50) and anti-CD3 PerCP/Cyanine5.5 (cat. number 100218, 1:150), which were all purchased from BioLegend. Next, the cells were fixed, resuspended in PBS, and analyzed on a BD FACS Canto™ flow cytometer. Gating strategies are showed in Supplementary Fig. 3.

### Enzyme-linked immunospot (ELISPOT) assay

The cellular immune responses of vaccinated mice were assessed using mouse IFN-γ and IL-2 ELISpot Kits (MabTech, Sweden) according to the manufacturer’s protocols. In brief, a total of 1 × 10^5^ splenocytes from immunized mice were stimulated with fourteen peptides generated from RVFV Gn and Gc proteins that encompassed the immunodominant epitopes identified in BALB/c mice (2 µg/mL)^58^ and seeded in precoated ELISpot plates for 16 h at 37 °C with 5% CO_2_. Then the plates were washed five times with PBS and incubated with a biotin-conjugated detection antibody at room temperature for 1 h. After washing, the plates were incubated with streptavidin-HRP for 1 h at room temperature. The cells were rinsed again, and the spots were formed with TMB substrate. Finally, the plates were rinsed thoroughly with deionized water, and the spots were counted on an AID ELISPOT reader (AID GmbH, Strassberg, Germany).

The cellular immune responses of vaccinated macaques were assessed using monkey IFN-γ, IL-2 and human IL-4 ELISpot kits (MabTech) according to the manufacturer’s protocols. PBMCs from immunized macaques were incubated with the peptide pool of 15-mer peptides with 11 overlapping amino acids for RVFV Gn and Gc proteins (2 μg/mL) in precoated 96-well ELISPOT plates with anti-monkey IFN-γ, anti-monkey IL-2 or anti-human IL-4 antibodies for 24 h at 37 °C. Then, the plates were washed five times with PBS and incubated with biotinylated anti-monkey IFN-γ, IL-2 or anti-human IL-4 detection antibody at room temperature for 2 h. The plates were washed five times with PBS before adding streptavidin-HRP. The TMB substrate was used to develop spots. Spots were counted and analyzed on an AID ELISPOT reader (AID GmbH).

To measure antigen-specific memory B cells of vaccinated macaques, a Human IgG ELISpot BASIC kit (HRP) was used. PBMCs (2 × 10^6^ cells/well) were incubated with R848 (1 μg/mL) and human IL-2 (10 ng/mL) at 37 °C for 4 days to differentiate MBCs into antibody-secreting cells. ELISpot plates were prewetted with 35% ethanol, rinsed and coated overnight with 100 μL/well anti-human IgG capture antibody (15 μg/mL) at 4 °C. After 4 days of incubation, cells were harvested, washed, counted, and adjusted to the designated concentration. The coated ELISpot plates were washed five times with PBS, blocked for 1 h at room temperature and emptied. Then, the cell suspension was added to the plates and incubated in a CO_2_ incubator at 37 °C for 20 h. After that, the plates were washed five times with PBS and incubated with biotinylated Gn and Gc proteins or biotinylated anti-human IgG detection antibody at room temperature for 2 h. Following washing, streptavidin-HRP was added to the plates for 1 h. Spots corresponding to antigen-specific MBCs were developed with TMB substrate and finally counted and analyzed on an AID ELISPOT reader (AID GmbH).

### Viral RNA extraction and RT‒PCR

The viral loads in the spleen and liver of challenged mice were determined by RT-PCR. In brief, the spleen and liver were collected and homogenized. Viral RNA was extracted with an RNeasy Mini Kit (Qiagen, Germany) following the manufacturer’s instructions. Reverse transcription was conducted using PrimeScript™ RT Master Mix (Takara, Japan). qPCR was performed with the following primers and probes described previously: forward primer 5′-GAAAATTCCTGAAACACATGG-3′, reverse primer 5′-ACTTCCTTGCATCATCTGATG-3′ and probe FAM- CAATGTAAGGGGCCTGTGTGGACTTGTG-BHQ1-3′^59^. Reactions were run on a QuantStudio 3 instrument (Applied Biosystems). Plasmid including a partial sequence of the RVFV L gene was serially diluted and used to generate the standard curve.

### Statistical analysis

Statistical analyses were performed using Prism 8.0 (GraphPad Software Inc., CA, USA). Data are displayed as the mean ± SEM. Differences in antibody titers, T-cell responses, GC B cell and Tfh cell responses were calculated by one-way ANOVA with Dunnett’s multiple comparisons test. A two-sided *p* < 0.05 was considered statistically significant.

### Reporting summary

Further information on research design is available in the Nature Research Reporting Summary linked to this article.

### Supplementary information

Supplementary materials

Reporting Summary

## Data Availability

All data that support the findings of this study are available from the corresponding author upon reasonable request.
