# Author Correction: A Rift Valley fever mRNA vaccine elicits strong immune responses in mice and rhesus macaques

**DOI:** 10.1038/s41541-023-00780-1

**Published:** 2023-11-28

**Authors:** Ting Bian, Meng Hao, Xiaofan Zhao, Chuanyi Zhao, Gang Luo, Zhendong Zhang, Guangcheng Fu, Lu Yang, Yi Chen, Yudong Wang, Changming Yu, Yilong Yang, Jianmin Li, Wei Chen

**Affiliations:** 1 Laboratory of Vaccine and Antibody Engineering, Beijing Institute of Biotechnology, 100071 Beijing, China; 2 Institute of Veterinary Medicine, Jiangsu Academy of Agricultural Sciences, Key Laboratory of Veterinary Biological Engineering and Technology, Ministry of Agriculture and Rural Affairs, Nanjing, China; 3 Frontier Biotechnology Laboratory, Zhejiang University-Hangzhou Global Scientific and Technological Innovation Center, Hangzhou, China; 4 School of Biotechnology, Jiangsu University of Science and Technology, Zhenjiang, China

**Keywords:** RNA vaccines, Virology

Correction to: *npj Vaccines* 10.1038/s41541-023-00763-2, published online 27 October 2023

In the original version of this article, an affiliation was missing for Ting Bian author. This has now been corrected in both the HTML and PDF version of the article.

