# Supplementary material for: A Rift Valley fever mRNA vaccine elicits strong immune responses in mice and rhesus macaques
Source: NPJ Vaccines. 2023 Oct 27;8:164. doi: 10.1038/s41541-023-00763-2 (PMC10611786; doi:10.1038/s41541-023-00763-2)
Supplement: Supplementary file 1 — Supplementary materials [file 41541_2023_763_MOESM1_ESM.pdf]

Supplementary Materials

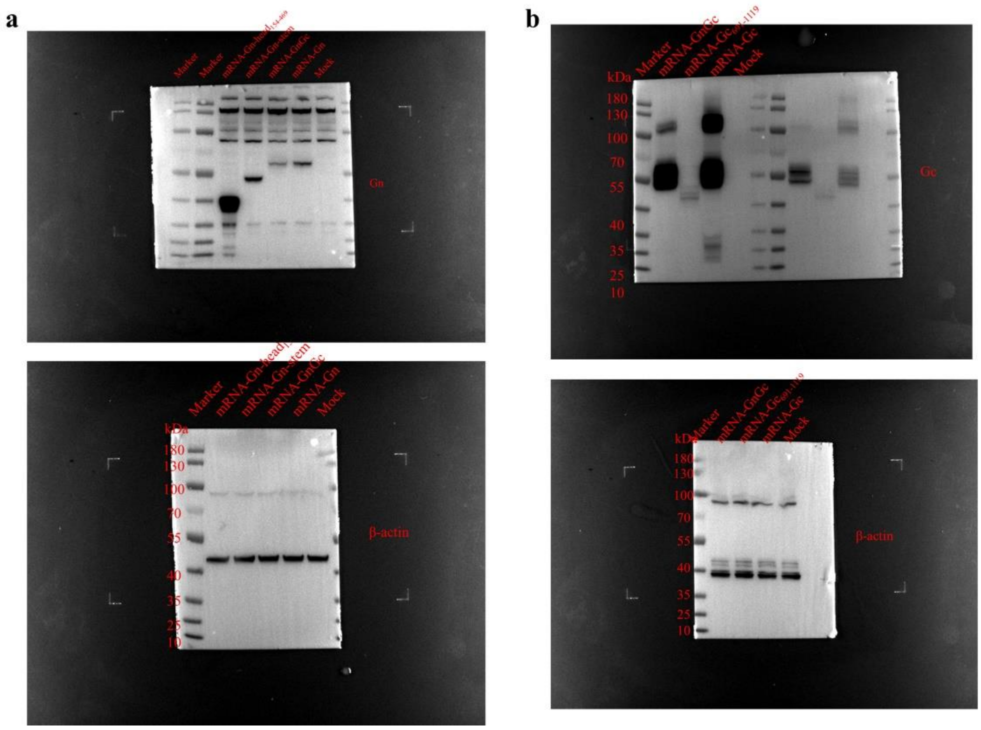

Supplementary Fig. 1. The full and un-cropped images of Fig.1b(a) and Fig.1c(b).

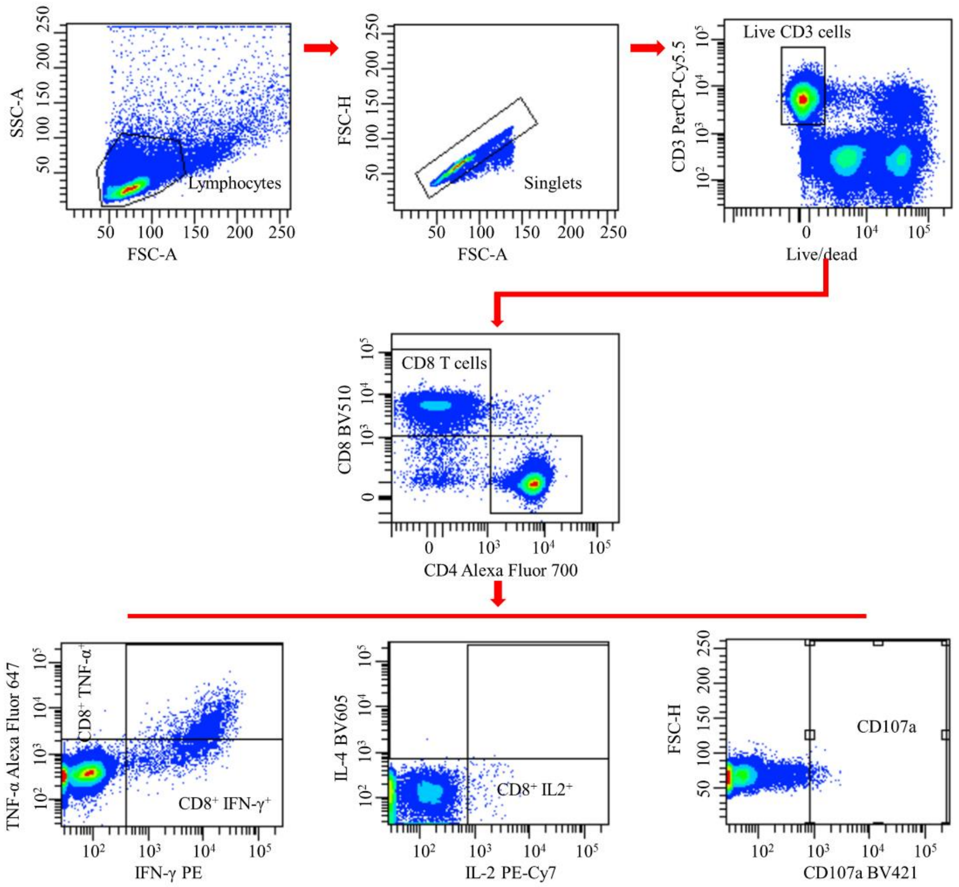

Supplementary Fig. 2. Representative gating strategy for intracellular cytokine staining. Groups of BALB/c mice (n = 6) were immunized intramuscularly with 5 μg of mRNA-Gn-head$_{154-469}$, mRNA-Gn-stem, mRNA-Gn, mRNA-Gc$_{691-1119}$, mRNA-Gc, mRNA-GnGc or a placebo and boosted with an equivalent dose 14 days later. Two weeks after booster immunization, the mice were sacrificed. Then the splenocytes were collected and stimulated with RVFV Gn and Gc peptides. After staining with different fluorochrome-conjugated antibodies, flow cytometric analysis was carried out to assess multifunctional cytokine-positive T cell subsets.

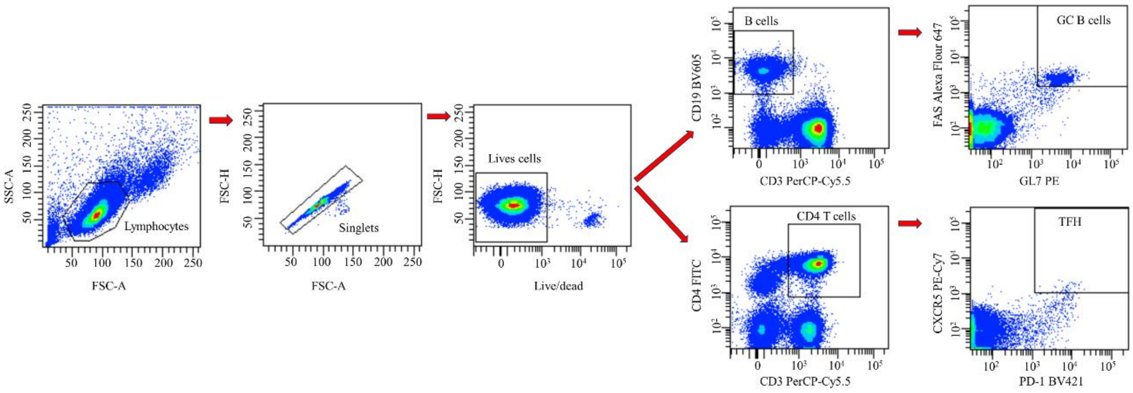

Supplementary Fig. 3. Representative gating strategy for GC B cells and Tfh cells. Groups of BALB/c mice (n = 6) were immunized intramuscularly with 5 μg of mRNA-Gn-head$_{154-469}$, mRNA-Gn-stem, mRNA-Gn, mRNA-Gc$_{691-1119}$, mRNA-Gc, mRNA-GnGc or a placebo and boosted with the same dose at a 14-day interval. Ten days after booster immunization, the mice were sacrificed, and the inguinal lymph nodes were collected for flow cytometric analyses. GC B cells were defined as live CD3^{-}CD19^{+}GL7^{+}Fas^{+} cells, and Tfh cells were defined as live CD3^{+}CD4^{+}PD-1^{+}CXCR5^{+} cells.
