# Supplementary material for: A Rift Valley fever mRNA vaccine elicits strong immune responses in mice and rhesus macaques
Source: NPJ Vaccines. 2023 Oct 27;8:164. doi: 10.1038/s41541-023-00763-2 (PMC10611786; doi:10.1038/s41541-023-00763-2)
Supplement: Supplementary file 2 — Reporting Summary [file 41541_2023_763_MOESM2_ESM.pdf]

nature portfolio

NPJVACCINES-03140
Corresponding author(s): Wei Chen
Jianmin Li
Yilong Yang

Last updated by author(s): Aug 27, 2023

Reporting Summary

Nature Portfolio wishes to improve the reproducibility of the work that we publish. This form provides structure for consistency and transparency in reporting. For further information on Nature Portfolio policies, see our Editorial Policies and the Editorial Policy Checklist.

Statistics

For all statistical analyses, confirm that the following items are present in the figure legend, table legend, main text, or Methods section.

n/a	Confirmed	
☐	☒	The exact sample size (*n*) for each experimental group/condition, given as a discrete number and unit of measurement
☐	☒	A statement on whether measurements were taken from distinct samples or whether the same sample was measured repeatedly
☐	☒	The statistical test(s) used AND whether they are one- or two-sided<br>*Only common tests should be described solely by name; describe more complex techniques in the Methods section.*
☒	☐	A description of all covariates tested
☒	☐	A description of any assumptions or corrections, such as tests of normality and adjustment for multiple comparisons
☐	☒	A full description of the statistical parameters including central tendency (e.g. means) or other basic estimates (e.g. regression coefficient) AND variation (e.g. standard deviation) or associated estimates of uncertainty (e.g. confidence intervals)
☒	☐	For null hypothesis testing, the test statistic (e.g. *F*, *t*, *r*) with confidence intervals, effect sizes, degrees of freedom and *P* value noted<br>*Give P values as exact values whenever suitable.*
☒	☐	For Bayesian analysis, information on the choice of priors and Markov chain Monte Carlo settings
☒	☐	For hierarchical and complex designs, identification of the appropriate level for tests and full reporting of outcomes
☒	☐	Estimates of effect sizes (e.g. Cohen's *d*, Pearson's *r*), indicating how they were calculated

Our web collection on statistics for biologists contains articles on many of the points above.

Software and code

Policy information about availability of computer code

Data collection	no software was used.
Data analysis	GraphPad Software Prism 8.0<br>FlowJo v10 software<br>Microsoft Office 2019

For manuscripts utilizing custom algorithms or software that are central to the research but not yet described in published literature, software must be made available to editors and reviewers. We strongly encourage code deposition in a community repository (e.g. GitHub). See the Nature Portfolio guidelines for submitting code & software for further information.

Data

Policy information about availability of data

All manuscripts must include a data availability statement. This statement should provide the following information, where applicable:

- Accession codes, unique identifiers, or web links for publicly available datasets
- A description of any restrictions on data availability
- For clinical datasets or third party data, please ensure that the statement adheres to our policy

All data that support the findings of this study are available from the corresponding author upon reasonable request.

Research involving human participants, their data, or biological material

Policy information about studies with [human participants or human data](). See also policy information about [sex, gender (identity/presentation), and sexual orientation]() and [race, ethnicity and racism]().

Reporting on sex and gender	*Use the terms sex (biological attribute) and gender (shaped by social and cultural circumstances) carefully in order to avoid confusing both terms. Indicate if findings apply to only one sex or gender; describe whether sex and gender were considered in study design; whether sex and/or gender was determined based on self-reporting or assigned and methods used.<br>Provide in the source data disaggregated sex and gender data, where this information has been collected, and if consent has been obtained for sharing of individual-level data; provide overall numbers in this Reporting Summary. Please state if this information has not been collected.<br>Report sex- and gender-based analyses where performed, justify reasons for lack of sex- and gender-based analysis.*
Reporting on race, ethnicity, or other socially relevant groupings	*Please specify the socially constructed or socially relevant categorization variable(s) used in your manuscript and explain why they were used. Please note that such variables should not be used as proxies for other socially constructed/relevant variables (for example, race or ethnicity should not be used as a proxy for socioeconomic status).<br>Provide clear definitions of the relevant terms used, how they were provided (by the participants/respondents, the researchers, or third parties), and the method(s) used to classify people into the different categories (e.g. self-report, census or administrative data, social media data, etc.)<br>Please provide details about how you controlled for confounding variables in your analyses.*
Population characteristics	*Describe the covariate-relevant population characteristics of the human research participants (e.g. age, genotypic information, past and current diagnosis and treatment categories). If you filled out the behavioural & social sciences study design questions and have nothing to add here, write "See above."*
Recruitment	*Describe how participants were recruited. Outline any potential self-selection bias or other biases that may be present and how these are likely to impact results.*
Ethics oversight	*Identify the organization(s) that approved the study protocol.*

Note that full information on the approval of the study protocol must also be provided in the manuscript.

Field-specific reporting

Please select the one below that is the best fit for your research. If you are not sure, read the appropriate sections before making your selection.

☒ Life sciences ☐ Behavioural & social sciences ☐ Ecological, evolutionary & environmental sciences

For a reference copy of the document with all sections, see [nature.com/documents/nr-reporting-summary-flat.pdf]()

Life sciences study design

All studies must disclose on these points even when the disclosure is negative.

Sample size	The sample size of animal studies was chosen taking into account previous works in this area.<br>Calvo-Pinilla, E. et al. A protective bivalent vaccine against Rift Valley fever and bluetongue. NPJ Vaccines 5, 70, doi:10.1038/s41541-020-00218-y (2020).<br>Huang, Q. et al. A single-dose mRNA vaccine provides a long-term protection for hACE2 transgenic mice from SARS-CoV-2. Nat Commun 12, 776, doi:10.1038/s41467-021-21037-2 (2021).<br>Zhang, N. N. et al. A Thermostable mRNA Vaccine against COVID-19. Cell 182, 1271-1283 e1216, doi:10.1016/j.cell.2020.07.024 (2020).
Data exclusions	No data have been excluded from the analyses
Replication	All the analyses have been replicated
Randomization	Animals were allocated in experimental groups randomly
Blinding	The researchers were not blinded to the study.

Reporting for specific materials, systems and methods

We require information from authors about some types of materials, experimental systems and methods used in many studies. Here, indicate whether each material, system or method listed is relevant to your study. If you are not sure if a list item applies to your research, read the appropriate section before selecting a response.

Materials & experimental systems

n/a	Involved in the study	
☐	☒	Antibodies
☐	☒	Eukaryotic cell lines
☒	☐	Palaeontology and archaeology
☐	☒	Animals and other organisms
☒	☐	Clinical data
☒	☐	Dual use research of concern
☒	☐	Plants

Methods

n/a	Involved in the study	
☒	☐	ChIP-seq
☐	☒	Flow cytometry
☒	☐	MRI-based neuroimaging

Antibodies

Antibodies used

anti-mouse CD16/32: S17011E clone, BioLegend, Cat number: 156604
anti-mouse CD19 Brilliant Violet 605™: 6D5 clone, BioLegend, Cat number:115539
anti-mouse GL7 PE: GL7 clone, BioLegend, Cat number:144608
anti-mouse Fas AF647: SA367H8 clone, BioLegend, Cat number:152620
anti-mouse CD4 FITC: RM4-5 clone, BioLegend, Cat number:100510
anti-mouse PD-1 Brilliant Violet 421™: 29F.1A12 clone, BioLegend, Cat number:135217
anti-mouse CXCR5 PE/Cyanine7 : L138D7 clone, BioLegend, Cat number:145516
anti-mouse CD3 PerCP/Cyanine5.5: 17A2 clone, BioLegend, Cat number:100218
anti-mouse CD4 Alexa Fluor 700: RM4-5 clone, BioLegend, Cat number:100536
anti-mouse CD19 APC/Cyanine7: 6D5 clone, BioLegend, Cat number:115530
anti-mouse CD8a Brilliant Violet 510™: 53-6.7 clone, BioLegend, Cat number:100751
anti-mouse CD107a Brilliant Violet 421™: 1D4B clone, BioLegend, Cat number:121617
anti-mouse IFN-γ PE: XMG1.2 clone, BioLegend, Cat number:505808
anti-mouse TNF-α Alexa Fluor 647: MP6-XT22 clone, BioLegend, Cat number:506314
anti-mouse IL-2 PE/Cyanine7: JES6-5H4 clone, BioLegend, Cat number:503832
Goat Anti-Mouse IgG H&L (HRP), Abcam, Cat number: ab6789
Goat Anti-Monkey IgG H&L (HRP), Abcam, Cat number: ab112767
Goat Anti-Human IgG Fc (HRP), Abcam, Cat number: ab97225
HRP Mouse monoclonal [AC-15] to beta Actin, Abcam, Cat number: ab49900
Mouse IFN-gamma ELISpotPLUS kit (HRP), Mabtech,Cat number: 321-4HPT-2
Mouse IL-2 ELISpotBASIC kit (HRP), Mabtech,Cat number: 3441-2H
Monkey IFN-gamma ELISpotPLUS kit (HRP), strips,Mabtech,Cat number:3421M-4HST-2
Human IL-4 ELISpotPLUS kit (HRP), Mabtech,Cat number: 3410-4HPW-2
ELISpot Plus: Monkey IL-2 (HRP), Mabtech,Cat number: 3445M-4HPW-2
Human IgG ELISpotBASIC kit (HRP), Mabtech,Cat number: 3850-2H

Validation

We follow the manufactures' instruction to use the above listed antibodies . All antibodies work well.

Eukaryotic cell lines

Policy information about cell lines and Sex and Gender in Research

Cell line source(s)

Hela cells(ATCC) were were used in the in vitro transfection assay; Vero E6 cells(ATCC) were used in RVFV neutralization assay; Expi293F cells(ThermoFisher) were used in Gn and Gc proteins expression experiment.

Authentication

Authenticated by vendors.

Mycoplasma contamination

ATCC cell lies are guaranteed to by mycoplasma negative. We used standard antobiotics treatment in media as recommended by the vendors.

Commonly misidentified lines
(See ICLAC register)

NA

Animals and other research organisms

Policy information about studies involving animals; ARRIVE guidelines recommended for reporting animal research, and Sex and Gender in Research

Laboratory animals

Female BALB/c mice, aged 6-8 weeks, used in this study were purchased from SPF (Beijing) Biotechnology Co., Ltd. (Beijing, China); Interferon-α/β receptor-deficient (IFNAR(−/−)) A129 mice were preserved and housed in the animal facility of the Animal Center, Beijing Institute of Biotechnology.
Five healthy rhesus macaques (2 females, 3 males, between 5-6 years old) used for vaccine immunogenicity analysis were purchased and housed at the Beijing Institute of Xieerxin Biology Resource.

Wild animals

The study did not involve wild animals.

Reporting on sex	BALB/C mice used in the immunological evaluation of the mRNA vaccines were all female. Equal numbers of both male and female Interferon-α/β receptor-deficient A129 mice were used in RVFV challenge experiment. Two females and three males rhesus macaques were used in this work.
Field-collected samples	The study did not involve field-collected samples.
Ethics oversight	All animal experimental protocols were approved by the Animal Care and Use Committee of Beijing Institute of Biotechnology, China (Permit number for mouse experiments: IACUC-SWGCYJS-2021-006, Permit number for rhesus macaque experiments: E20220615) in strict accordance with the Guide for the Care and Use of Laboratory Animals of the People's Republic of China.

Note that full information on the approval of the study protocol must also be provided in the manuscript.

Flow Cytometry

Plots

Confirm that:

☒ The axis labels state the marker and fluorochrome used (e.g. CD4-FITC).

☒ The axis scales are clearly visible. Include numbers along axes only for bottom left plot of group (a 'group' is an analysis of identical markers).

☒ All plots are contour plots with outliers or pseudocolor plots.

☒ A numerical value for number of cells or percentage (with statistics) is provided.

Methodology

Sample preparation	BALB/c mice (n = 6) were immunized intramuscularly with 5 μg of mRNA-Gn-head154-469, mRNA-Gn-stem, mRNA-Gn, mRNA-Gc691-1119, mRNA-Gc, mRNA-GnGc or a placebo and boosted with the same dose at a 14-day interval. Ten days after booster immunization, the mice were sacrificed, and the inguinal lymph nodes were collected.The draining inguinal LNs were homogenized and filtered through a 70-mm cell strainer, and 2×106 cells were stained with the viability dye Near-IR and blocked with anti-mouse CD16/32 (S17011E clone, BioLegend, USA).Then, the cells were stained with a mixture of monoclonal antibodies, including anti-CD19 Brilliant Violet 605™ (6D5 clone), anti-GL7 PE (GL7 clone), anti-Fas APC (SA367H8 clone), anti-CD4 FITC (RM4-5 clone), anti-PD-1 Brilliant Violet 421™ (29F.1A12 clone), anti-CXCR5 PE/Cyanine7 (L138D7 clone) and anti-CD3 PerCP/Cyanine5.5 (17A2 clone), which were all purchased from BioLegend. Next, the cells were fixed, resuspended in PBS, and analysed on a BD FACS Canto™ flow cytometer..<br>For intracellular cytokine staining assay<br>splenocytes from immunized mice were seeded in 24-well plates (2 × 106 cells/well) and stimulated with fourteen peptides derived from RVFV Gn and Gc proteins (2 μg/mL) , together with BD GolgiStop™ (BD Biosciences) for 8 h at 37°C. The cells were harvested, incubated in live/dead near IR (Thermo Fisher) and blocked with anti-CD16/32 (S17011E clone, BioLegend). Following a wash in PBS, cells were stained for 30 min with a mixture of anti-mouse antibodies purchased from BioLegend, including CD3 PerCP/Cyanine5.5 (17A2 clone), CD4 Alexa Fluor 700 (RM4-5 clone), CD19 APC/Cyanine7 (6D5 clone), CD8a Brilliant Violet 510™ (53-6.7 clone), and CD107a Brilliant Violet 421™ (1D4B clone). After a wash in PBS, cells were fixed and permeabilized with Cytofix/Cytoperm (BD Biosciences), washed with Perm/Wash buffer (BD Biosciences), and stained for 30 min with a mixture of anti-mouse antibodies purchased from BioLegend, including IFN-γ PE (XMG1.2 clone), TNF-α APC (MP6-XT22 clone) and IL-2 PE/Cyanine7 (JES6-5H4 clone). Finally, cells were washed and resuspended in PBS prior to the acquisition of data on a FACS Canto™ flow cytometer (BD Biosciences).
Instrument	FACS Canto™ flow cytometer (BD Biosciences)
Software	Data were analyzed with FlowJo software V10
Cell population abundance	N/A
Gating strategy	The gating strategy is consistent with our previous work. Bian, T. et al. Single-dose of a replication-competent adenovirus-vectored vaccine provides sterilizing protection against Rift Valley fever virus challenge. Front Immunol 13, 907675, doi:10.3389/fimmu.2022.907675 (2022).

☒ Tick this box to confirm that a figure exemplifying the gating strategy is provided in the Supplementary Information.
